# Mitochondria as a Therapeutic Target for Burn Injury

**DOI:** 10.3390/biom16040520

**Published:** 2026-04-01

**Authors:** Igor Prudovsky, Anyonya R. Guntur, Joseph Rappold, Damien Carter

**Affiliations:** 1 Center for Molecular Medicine, MaineHealth Institute for Research, Scarborough, ME 04074, USA; anyonya.guntur@mainehealth.org (A.R.G.); jfrappold@yahoo.com (J.R.); damien.carter@mainehealth.org (D.C.); 2 Tufts University School of Medicine, Tufts University, Boston, MA 02111, USA; 3 Graduate School of Biomedical Sciences and Engineering, University of Maine, Orono, ME 04469, USA; 4 Department of Surgery, MaineHealth Maine Medical Center, Portland, ME 04102, USA

**Keywords:** burn injury, mitochondria, DAMPs, mitophagy, edema, SIRS

## Abstract

Severe burn injury results in systemic inflammation, edema, multiple organ disorder and muscle wasting. These events are provoked by the massive dysfunction of mitochondria not only in the burned skin but also in muscles and internal organs, which is induced by the release of damage-associated molecular patterns and catecholamines. Dysfunctional mitochondria are characterized by increased ROS production and the release of mitochondrial DNA, which lead to enhanced expression of proinflammatory cytokines. Mitochondria present a key target for treatment of severe burns, and various pharmacological approaches are being developed to protect normal mitochondrial functions after burn injury.

## 1. Severe Burn and Mitochondria

Despite remarkable improvements in burn care over the past 70 years, severe burns that are refractory to current resuscitation practices continue to challenge burn care providers and remain a common contributor to burn-related mortality and morbidity. This is primarily because of inflammatory complications resulting from an overstimulated and dysfunctional immune response [1,2]. The loss of microvascular integrity is central to burn pathophysiology and leads to large fluid-volume resuscitation requirements, ultimately precipitating widespread tissue edema and end organ dysfunction [3]. Deaths after severe trauma are related to trauma-induced metabolic stress, leading to systemic inflammatory response syndrome (SIRS), endotheliopathy, sepsis, organ failure and mortality [4]. Mitochondrial dysfunction is central to metabolic stress and is the common pathway for organ injury and SIRS after trauma [5].

Mitochondria are powerhouses for multiple intracellular processes, including ATP generation, programmed cell death, and intracellular signal transduction [6]. Recent studies have recognized mitochondria as critical to the initiation of SIRS [5,6]. Indeed, mitochondria-derived damage-associated molecular patterns (DAMPs) initiate SIRS by acting on transmembrane receptors (e.g., Toll-like receptors (TLR)), resulting in the activation of inflammatory signaling in the cells [7]. Disorderly mitochondrial activity interferes with normal cellular function in various cell types [5,7]. Evidence of mitochondrial dysfunction after injury has been associated with renal, liver and respiratory failure in animal models [8,9]. Conversely, normal mitochondrial function can support orderly intracellular conditions and may prevent the development of SIRS-related complications [10,11]. Mitophagy (organized degradation of dysfunctional mitochondria) and mitochondrial biogenesis (generation of new mitochondria) are critical processes that maintain normal mitochondrial function in all cells [10]. The role of mitophagy may be especially important in the context of trauma associated with the damage of mitochondria and release of pro-inflammatory mitochondrial DAMPs. Mitophagy could ensure the organized disposal of damaged mitochondria and prevent the export of DAMPs to the extracellular milieu. Recent studies have begun to elucidate the signaling mechanisms that sustain orderly mitophagy and biogenesis [12,13], which will help the development of potential agents to support normal mitochondrial function. Effective modulation of the immune response to injury to avoid the deleterious effects of SIRS and related organ dysfunction remains an elusive target in trauma research [14,15]. In this context, targeting mitochondria behavior—given its importance to cell and organ function during physiologic stress—represents a novel approach to the challenge. As mitochondria are a source of several potent DAMPs released into circulation after injury, reducing mitochondrial damage and improving bioenergetics would be expected to modulate the immune response and reduce organ dysfunction [16,17].

## 2. Burn-Induced Mitochondrial Dysfunction in Specific Tissues and Organs

Electron microscopy studies have demonstrated that severe burns result in rapid and dramatic changes in fine mitochondrial morphology, even in organs that have not directly experienced burn injury, such as the heart, gut, and liver. Burns induce swelling of mitochondria, a decrease in mitochondrial matrix density and deterioration of cristae [18,19,20,21,22,23,24]. Burn-induced changes of mitochondrial function in various organs are also well documented.

### 2.1. Skeletal Muscles

Burn injury results in a significantly reduced rate of ATP production in mouse hind limb muscles [25]. Cree et al. reported a decrease in the mitochondrial oxidation of palmitate and pyruvate in the skeletal muscle of burn patients [26]. Sidossis and colleagues [27] performed high-resolution respirometry on muscle fibers isolated from the spinotrapezius muscles of mice at different time points after severe spinal burn. They demonstrated a strong decrease in mitochondrial respiration coupling to ATP production and in maximal respiratory capacity within 3 h after burn. By 10 days, these parameters were restored to normal levels. In contrast, the fibers isolated from a distant muscle, the quadriceps, exhibited significantly decreased respiration only 10 days after burn. At 11–21 days the mitochondria in the quadricep muscles of burned patients exhibited decreased oxidation capacity and cytochrome oxidase activity, and increased uncoupling. In the muscles of children who are victims of burn injury, decreased oxidative capacity was observed as late as 1 year after burn, and mitochondrial coupling control remained diminished even after 2 years [28]. The muscles of burn patients show an increase in markers of the mitochondrial unfolded protein response (mt-UPR) pathway and the upregulation of mitochondrial specific proteases LONP1 and CLPP and mitochondrial translocases TOM40, TIM23 and TIM17B [29]. This finding reflects the increased mitochondrial proteolysis and compensatory import of proteins to mitochondria [29].

Muscle wasting is a notable organism response to severe burn. It is a protracted process which results in the loss of significant portions of muscle mass [30]. While initially muscle wasting is needed to provide energy and amino acids for wound repair, in the long term it may become a detrimental process slowing down the healing of the organism after burn trauma. Severe burn has been shown to result in the increased expression of uncoupling proteins UCP2 and UCP3 in skeletal mitochondria [31,32]. The uncoupling of mitochondrial respiration in skeletal muscle causes increased production of harmful ROS [33]. Mitochondrial dysfunction leads to the apoptotic death of a portion of muscle fibers [34].

### 2.2. Heart

Like skeletal muscle, severe burn strongly suppresses the activity of mitochondria in the myocardium. Experiments on rodent models of severe burn have shown that burn decreases the production of mitochondrial ATP [22,35] and activity of mitochondrial electron transfer chain complexes except complex II [35]. Similar results were obtained in the porcine model of severe burn [36]. The coupling efficiency of heart mitochondria is significantly decreased after burn [37]. Wen and colleagues demonstrated that severe burn decreases the abundance of mtDNA in rat myocardium and expression of all mtDNA-coded genes [35]. Szczesny et al. [37] also found a decrease in mtDNA integrity in the hearts of severely burned mice.

### 2.3. Lungs

Szczesny and colleagues [37] found, at 3h after severe burn, a marginal decrease in mitochondrial respiration, ATP production and proton leakage in the lung mitochondria of mice; however, by 10 days these characteristics significantly exceeded control levels. The same study demonstrated a transient decrease in lung mitochondrial volume followed by an increase above control at later time points after the burn. At the same time, burn induced a rapid decrease in lung mtDNA integrity, which was sustained until at least 10 days.

### 2.4. Liver

In mouse liver, severe burn causes a lasting decrease in mitochondrial respiration and an increase in uncoupling [38]. A study of isolated rat hepatic mitochondria demonstrated the suppression of cytochrome b and c activities as early as 30 min after the burn [39]. Burn also results in an increase in ROS production by rat liver mitochondria [40]. In the livers of high-fat-diet-fed mice, severe burn suppressed the contacts between mitochondria and endoplasmic reticulum, which resulted in a decrease in fatty acid β-oxidation [41]. Interestingly, a study by Bohanon et al. demonstrated that, in rat liver 24 h after burn, mitochondrial respiration coupled to ATP production was significantly higher than in the control, apparently reflecting liver adaptation [42]. In contrast, the ATP production and maximal respiratory capacity of mitochondria isolated from the liver of severely burned mice were significantly reduced both at 24 h after burn and were restored only at day 4 [37]. Unlike the heart, the livers of burned mice did not show a burn-induced decrease in mtDNA integrity [37].

### 2.5. Intestine and Pancreas

Severe burn results in mitochondrial uncoupling in rat intestinal epithelium [43]. It causes mitochondrial dysfunction [44], increases ROS production and suppresses the activity of mitochondrial complex III in mouse pancreatic islets [45].

### 2.6. Adipose

The changes induced by severe burn in adipose tissue deserve special consideration. Sidossis and colleagues reported that, 4 days after severe burn, the mitochondrial respiration in mouse white adipose was strongly enhanced due to the increase in mitochondrial mass [46]. Studies on rodent models of burn injury and in burn patients reported a burn-induced increase in brown adipose and the browning of white adipose. These changes are manifested already 24h after burn [47], and can last as long as 20 days [48]. The browning of white adipose is accompanied by the appearance of multilocular adipocytes (typical for brown adipose), high expression of mitochondrial uncoupling protein 1 (UCP1) and enhancement of uncoupled mitochondrial respiration [46,47,49]. Several days after burn injury, the weight of mouse white adipose significantly decreases, while that of brown adipose increases [50]. Interestingly, burn was shown to enhance, in mouse brown adipose, the expression of COX2, the enzyme responsible for production of proinflammatory prostanoids, and the treatment of mice with the COX2 inhibitor celecoxib diminished burn-induced UCP1 expression [51]. The burn-induced browning of white adipose is associated with increased lipolysis in adipose and steatosis in the liver. Genetic deletions of the proinflammatory cytokine IL6 and of UCP1 both diminished the burn-induced liver steatosis in mice [52]. The causative relation between burn-induced adipose browning and lipolysis needs further study. Indeed, the knockout of the major lipolytic enzyme, adipose triglyceride lipase (ATGL), was found to decrease adipose browning and hepatomegaly after burn [53].

In summary, multiple studies performed both in burn patients and animal models demonstrate that severe burns lead to dysfunction of mitochondria in various organs, including those distant from the burn sites (Table 1). However, studies involving more organs such as the kidney, bone and brain are still highly desirable. The response of blood cell mitochondria to burn also requires investigation because it can be directly involved in burn-induced inflammation. So far, we have been able to find only one study on this subject showing an increase in mitochondrial respiration in porcine lymphocytes 24–48 h after burn [54]. Adipose presents a special case in the response of tissues to severe burn: a strong increase in mitochondrial biogenesis is accompanied by the browning of adipose tissue, massive uncoupling of mitochondrial respiration and heat production. We suggest that this delayed hypermetabolic response of adipose to burn could be at least partially induced by earlier mitochondrial dysfunction in other organs. In general, the data reviewed in this chapter illustrates the importance of mitochondria as a potential target to achieve the stimulation of burn wound healing, prevention of edema, suppression of systemic inflammation and decrease in muscle wasting. Considering the systemic character of severe burn effects determined by humoral and nervous connections between different organs, we suggest that whole-organism mitochondria-based therapeutic approaches similar to those described in the final section of this review could have the highest therapeutic potential. The only obvious exception would be the topical treatment of the burn wound area. In any case, the earlier the treatment starts, the better the results can be expected to be.

## 3. Burn-Induced Release of DAMPs Induces Mitochondrial Dysfunction

The response of mitochondria in organs distant from burn sites raises a question about the mechanisms sending the message about burn injury. In early studies by Kremer et al., the intraperitoneal injection of burned skin extracts (“burn toxin”) caused the intramitochondrial vacuolization and loss of cristae [55,56]. Another early study has shown that the extracts of burned tissue impaired the respiratory control of isolated liver mitochondria [57]. In a recent study, the blood plasma from burn-injured mice induced the leakage of cytochrome c from mitochondria to cytoplasm and increased the ROS level in hepatocyte culture [58]. This data is in agreement with an earlier publication [59] showing that burn serum treatment of myoblast culture resulted in an increase in mitochondrial ROS and a decrease in cytochrome c oxidase activity as well as an increase in lipid oxidation and a decrease in antioxidant enzymes activities. Interestingly, the authors have shown that the mitochondrial damage caused by burn serum was dependent on the expression in the target cells of CD14, a co-receptor of proinflammatory TLRs, which bind DAMPs.

DAMPs, also known as alarmins, include a varied group of molecules that are normally located inside the cells but are released after cell damage and then induce an inflammatory reaction by binding to low-specificity receptors on target cells. Among DAMPs are many proteins, for example, HMBG1, S100a8, S100a9 and formylated mitochondrial proteins; mitochondrial DNA; oxidized phospholipids and cholesterol crystals; and ATP [60,61,62]. The release of some DAMPs, for example, HMGB1 and S100 family proteins, can happen not only as a result of cell destruction, but also from live stressed cells, through various ER–Golgi-independent mechanisms of nonclassical protein secretion [63].

Severe burns increase the blood level of HMGB1 in mice, and this effect is paralleled by the leakage of lung endothelium [64]. In vitro, HMBG1 treatment leads to the loss of endothelial monolayer integrity [64]. The early increase in blood HMGB1 in the porcine burn model also predicts the development of acute kidney injury [65]. Neutralizing anti-HMGB1 antibodies was demonstrated to alleviate burn-induced muscle loss in rats [66]. The HMGB1-containing extracellular vesicles isolated from the blood of severely burned mice induced the expression of proinflammatory cytokines in macrophage culture [67]. Interestingly, Coleman et al. reported that HMGB1 can form complexes with pro-IL1β in the plasma after burn injury [68]. Thus, two pro-inflammatory proteins devoid of signal peptide and unable to be secreted through ER–Golgi are released together during periods of stress. Similar to HMGB1, burns induce increases in S100a9 [69] and S100b [70] blood levels. The exosomes from the serum of burn injury patients also increased the permeability of endothelial monolayers in vitro [69].

In rodents, burn injury induced the upregulation of mtDNA blood content [37,71], and treatment with DNAse I not only removed mtDNA from the bloodstream, but also decreased the markers of systemic inflammation in burned animals [71]. The treatment of burn injured rats with epigallacatechine gallate decreased both mtDNA blood content and acute respiratory distress syndrome (ARDS), while the intravenous injection of mtDNA was reported to induce ARDS [72].

In most cases, DAMPs signal through TLR or RAGE (Receptor for Advanced Glycation End Products) low-specificity receptors [73]. Mitochondrial formylated proteins signal through the formyl methionine receptor (FPR), and ATP through purinergic receptors. The binding of DAMPs to receptors stimulates NFκB signaling, leading to the expression and release of proinflammatory cytokines and chemokines [60,61]. DAMPs also induce the dysfunction of mitochondria. Thus, HMBG1 causes mitochondrial oxidative damage [74]. S100a8/a9 induce mitochondrial dysfunction after ischemia/reperfusion [75]. Mitochondrial dysfunction is also one of the deleterious effects caused by extracellular ATP [76]. West and colleagues [77] found that signaling through TLR1, TLR2 and TLR4 augments ROS production by macrophage mitochondria. Sbai et al. [78] demonstrated that RAGE signaling mediated by TXNIP stimulates the association of amyloid-beta with mitochondria in microglial cells, resulting in mitochondrial dysfunction and oxidative stress. Amini et al. [79] showed that the activation of purinergic receptor P2X7 by extracellular ATP enhances ROS production by mitochondria, and this leads in turn to the ER–Golgi-independent release of proinflammatory cytokine IL1α.

**Figure 1:**
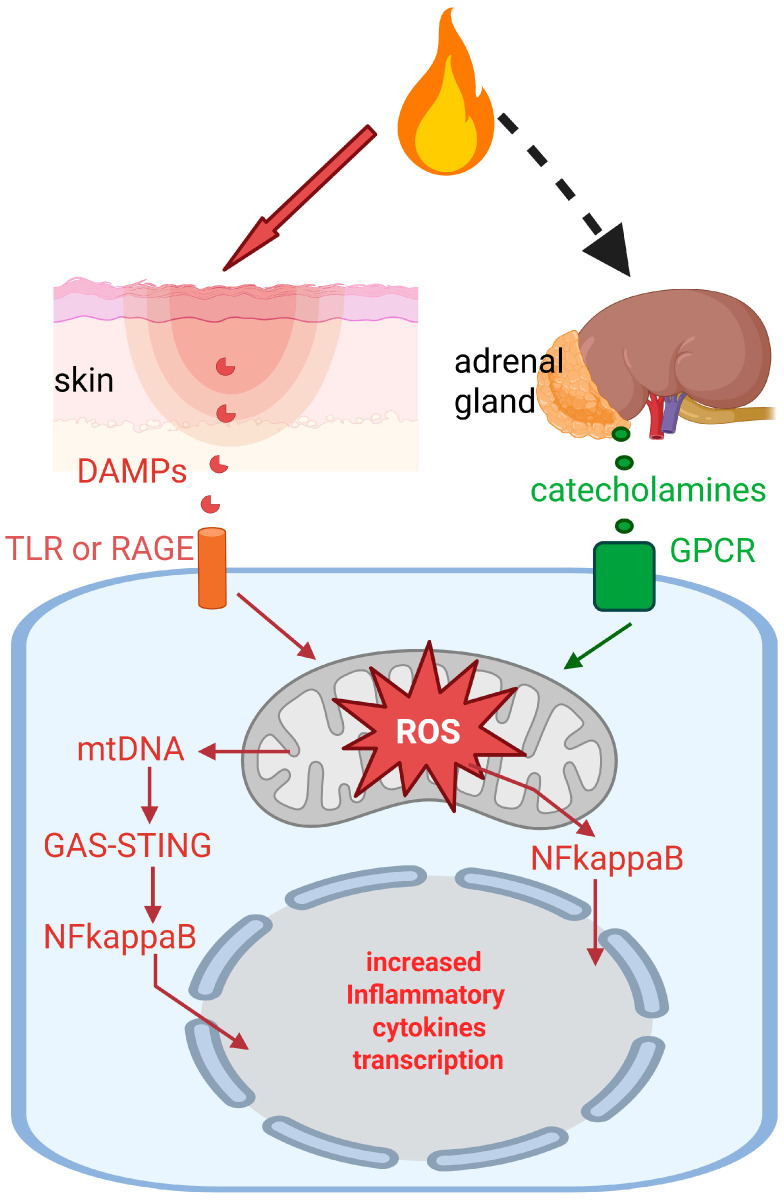
Induction of mitochondrial dysfunction by severe burn. At least two groups of factors transported by the bloodstream induce mitochondrial dysfunction and enhanced ROS production. DAMPs signal through low-specificity receptors, such as TLR or RAGE, and catecholamines, through G-protein-coupled receptors (GPCR). Enhanced ROS production and mtDNA leakage to cytoplasm result in the stimulation of NFκB signaling, leading to the increased transcription of genes coding for proinflammatory cytokines. Because of the ubiquitous expression of TLR, RAGE and GPCR, all organs are potential targets of burn-induced DAMPs and catecholamine signals. Created in BioRender. Prudovsky, I. (2026) https://app.biorender.com/illustrations/canvas-beta/696fd3db37b2eecc98ef0535 (accessed on 23 March 2026).

Collectively, these results indicate that DAMPs released from damaged tissues after burn injury and transported through the bloodstream induce mitochondrial dysfunction (Figure 1). This, in combination with the stimulation of the production and secretion of proinflammatory cytokines through DAMP receptor signaling, can result in organ dysfunction, systemic inflammation, hypermetabolism, muscle wasting and brown adipose hypertrophy. It remains to be determined which specific DAMPs are major detrimental agents in burn patients and whether a cumulative effect of several DAMPs occurs.

## 4. Catecholamines as Inducers of Mitochondria Dysfunction After Burn Injury

Besides DAMPs, other factors involved in burn-induced mitochondria dysfunction are catecholamines produced by the adrenal glands and massively released to the bloodstream after severe burn injury [80,81,82]. Enhanced catecholamine signaling through β-adrenergic receptors (Figure 1) can drastically increase Ca^2+^ levels in cytoplasm, followed by Ca^2+^ overload in mitochondria, which results in the uncoupling of oxidative phosphorylation, increased ROS production, damage to the mitochondrial structure and release of cytochrome c to cytoplasm [83,84]. The presence of catecholamine metabolites in target cells can also cause the mitochondrial damage [85]. It has been shown that β-blockers can alleviate the pathological effects of severe burn injury (for review see [86]).

## 5. Proinflammatory Effect of Cytoplasmic mtDNA at Burn

During cellular stress, mtDNA can be released into the cytoplasm, where it is sensed by cGAS protein and then initiates an inflammatory response through the adaptor STING protein. Moreover, oxidized cytoplasmic mtDNA can activate the NRLP3 inflammasome (for review see [87]). While, to our knowledge, the burn-induced release of mtDNA to cytoplasm has not yet been studied, Comish et al. [88] have recently demonstrated that, in burned rats, the increase in the mtDNA level in the bloodstream coincides with the upregulation of cGAS and STING proteins in lung tissue. These data indicate that the release of mtDNA to cytoplasm preceding its release to external milieu is another trigger of the burn-induced inflammatory response. This suggestion is supported by a recent work by Xie et al. [89], who found that C176, a specific inhibitor of STING, decreased the inflammatory cell infiltration and muscle wasting induced by burn injury in mice.

## 6. Potential Role of Mitophagy in Alleviating Burn Injury

Mitochondrial dysfunction caused by burn injury results in several problems for involved cells and the whole organism. First, it is the enhanced production of ROS; second, the leakage of mitochondrial DAMPs to cytoplasm and then to the extracellular space, where they propagate the systemic inflammatory response syndrome (SIRS); third, it is the leakage of cytochrome c to the cytoplasm, where it initiates apoptosis [90]. Damaged mitochondria undergo mitophagy that helps to prevent these undesirable effects of mitochondrial dysfunction by the sequestration of mitochondria in autophagosomes [12]. Autophagosomes containing mitochondria fuse with lysosomes, leading to proteolysis of their content.

**Figure 2:**
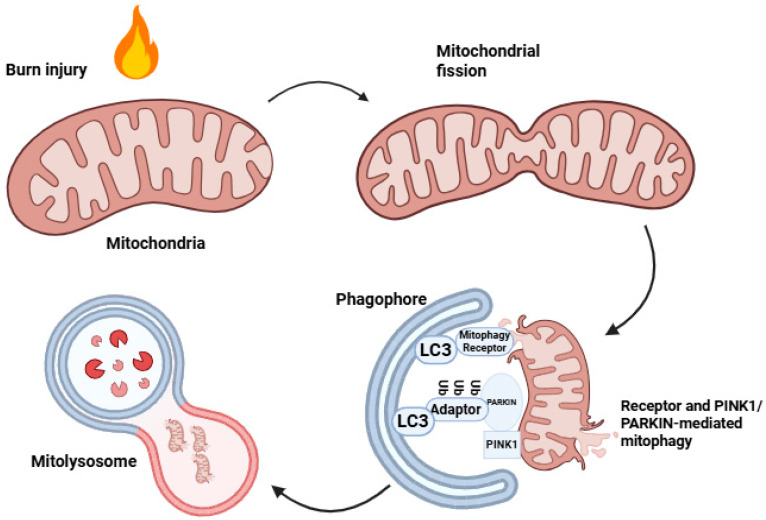
Burn-injury-induced mitophagy. Burn injury induces mitochondrial damage and fragmentation, resulting in mitochondrial fission and the segregation of dysfunctional mitochondrial segments. Damaged mitochondria are subsequently targeted for mitophagy through both receptor-mediated pathways and the PINK1/Parkin ubiquitin pathway. In this process, mitophagy receptors and adaptor proteins facilitate recruitment of LC3-positive phagophore membranes around injured mitochondria, promoting their sequestration within autophagic vesicles. These vesicles then fuse with lysosomes to form mitolysosomes, where mitochondrial components are degraded. This model illustrates the sequential steps linking burn-injury-associated mitochondrial stress to selective mitochondrial turnover by mitophagy. Created in Biorender. Guntur, A.R. (2026) https://app.biorender.com/illustrations/69bc04d271e1c07655790374 (accessed on 23 March 2026).

Mitophagy includes the classical PTEN-induced kinase 1 PINK1/PARKIN ubiquitination pathway, as well as receptor-mediated mitophagy pathways (Figure 2).

Receptor-mediated mitophagy involves proteins such as BCL-2/adenovirus E1B 19 kDa protein-interacting protein 3 (BNIP3), BCL-2/adenovirus E1B 19 kDa protein-interacting protein-3-like (BNIP3L), FUN14 domain-containing 1 (FUNDC1), BCL2-like 13 (BCL2L13), FK506-binding protein 38 (FKBP8), the activating molecule in Beclin-1-regulated autophagy protein 1 (AMBRA1), and Prohibitin 2 (PHB2) [91,92,93,94,95,96,97]. In addition, the more recently described mitochondrial-derived vesicle (MDV) pathway represents another mitochondrial quality control mechanism [98].

Mitophagy is activated in response to various stressors, including starvation or nutrient stress, ROS, hypoxia, genetic mutations, and burn injury. In particular, it has been demonstrated that ROS released by dysfunctional mitochondria induce the association of Parkin with the outer mitochondrial membrane [99].

The cellular response to stress at the molecular level is coordinated through the integrated stress response (ISR) pathway. In this pathway, cells recognize specific insults and, depending on the nature of the stress, invoke targeted adaptive responses. The most extensively studied factor in this process is Activating Transcription Factor 4 (ATF4). ATF4 is selectively translated in response to stress following phosphorylation of Ser51 on eukaryotic initiation factor 2α (eIF2α), while global protein translation is suppressed. In vertebrates, all four eIF2α kinases - heme-regulated inhibitor (HRI/EIF2AK1), protein kinase R (PKR/EIF2AK2), PKR-like endoplasmic reticulum kinase (PERK/EIF2AK3), and general control non-depressible 2 (GCN2/EIF2AK4) have been shown to regulate this process [100].

GCN2 is activated specifically in response to amino acid deficiency and metabolic stress and regulates proliferation upstream of ATF4, including the control of glutamine metabolism. In response to increased cellular stress, ATF4 can increase the import of amino acids, including proline, which is directly related to collagen synthesis [101]. HRI (EIF2AK1) has also been identified as a mitochondrial stress kinase and is activated by DELE1, a mitochondrial-derived protein that physically interacts with eIF2α kinases to promote their allosteric activation in response to mitochondrial stress [102].

Though there are several studies that have shown that there is hypercatabolism resulting in increased mitochondrial dysfunction in the skeletal muscle after burn injuries, there is not a clear indication of what happens with respect to mitophagy under these conditions. In a study looking at patients with greater than 30% of burn over total body surface [29], the authors identified increased oxygen consumption rates, increased mTORC1 signaling followed by activation of the mitochondrial unfolded protein response (mtUPR). There is also evidence suggesting that cGAS-STING-NFκB signaling is elevated and might be a cause of burn-induced muscle catabolism [89]. How these pathways are activated and controlled under burn conditions, and if any of the stress kinases and specifically if the mitochondrial stress-related kinase HRI is involved in regulating this process, still needs to be determined.

There is some evidence in the literature that there is an increase in cardiac oxidative stress with an increase in mitochondrial fission in rats with burn injury. The authors measured Opa1, which decreased with burn injury and is the mitochondrial fusion protein that generally has been shown to be involved in regulating mitochondrial homeostasis and dynamics. They also simultaneously showed that there is a significant decrease in Drp1 the mitochondrial fission GTPase [103,104], thereby dampening both fusion and fission in this model. It is again not clear, however, how this is related to mitophagy and if this dysfunction results in elevated stress responses that are mitochondrial-related and any of the mitochondrial stress responses are activated.

The effects of burn on mitochondria in liver cells have been studied, showing decreased lipid metabolism and mitochondrial dysfunction. The effects on mitochondrial turnover and dynamics again were not closely studied in this tissue, suggesting a potential therapeutic avenue that can be exploited in the future [105].

Guo et al. demonstrated an increase in both Parkin- and BNIP3-dependent mitophagy in the rat skin zones adjacent to the burned area and found that this effect depends on the HIF1α transcription factor [106]. Apparently, local hypoxia resulting from blood stasis, which is typical for these zones, triggers local mitophagy through HIF1α upregulation. Zhao et al. found that burn injury enhances Parkin/PINK1-mediated mitophagy in the skin [107].

Moderate to severe burn injury in humans and animal models results in increased intestinal tight junction barrier dysfunction. In mice, severe burn injury leads to elevated endoplasmic reticulum (ER) stress and autophagy, as evidenced by the increased expression of ER stress markers, including chaperone binding protein (BiP/GRP78), CCAAT/enhancer-binding protein, homologous protein (DDIT3/CHOP), and inositol-requiring enzyme 1 (IRE1)/X-box binding protein 1 splicing (XBP1), along with autophagy markers such as Beclin1. These findings support the conclusion that ER stress and autophagy activation contribute to intestinal barrier disruption in response to severe burn injury [108].

Although the effects of these changes on mitochondrial stress and other organellar stress responses were not directly examined, it is possible that the observed activation of stress-response pathways represents an initial protective adaptation aimed at counteracting severe-burn-injury-induced cellular damage with an adaptive response that may ultimately become insufficient to resolve the injury. We postulate that the sequence of events triggering increased mitophagy may follow this general progression.

## 7. Targeting Mitochondria to Ameliorate Burn Outcomes

The role of mitochondrial dysfunction in the outcomes of burn injury makes mitochondria an attractive target in studies aiming to develop novel pharmacological approaches to burn treatment. A group of studies assessed the effects of mitochondria-targeted antioxidant peptide SS-31 in animal burn models. SS-31 decreased the expression of markers of ER stress in burned mice [109]. It promoted the post-burn recovery of muscle by restoring the expression levels of mitochondrial enzymes, ATP production and mitochondrial coupling [110]. In rat models of burn injury, SS-31 ameliorated hepatic injury, decreased the release of mtDNA and suppressed the activity of the mtDNA-STING signaling pathway in Kupfer cells [111].

Mitochondria-targeted antioxidant Mito-TEMPO alleviated burn-injury-induced cardiac dysfunction in rats, decreasing cardiac inflammation and fibrogenesis [112]. Mito-TEMPO also strongly suppressed the burn-induced ROS increase in myocardium. ASMq, a natural herb compound, was shown to suppress burn wound progression in rats, and this effect could be related to the ability of ASMq to alleviate oxidative stress and prevent mitochondria-associated apoptosis [113]. The amino acid taurine is known for its antioxidant effect and ability to maintain mitochondrial function [114]. Taurine was shown to protect the activities of mitochondrial enzymes in the cardiomyocytes of burn-injured rats [115]. It promoted re-epithelization and hair re-growth in burned rat skin [116]. Taurine also exhibited anti-inflammatory effects in burned patients [117]. Coenzyme Q10, a cofactor in the mitochondrial electron transport chain, acting in its reduced form as a lipophilic antioxidant, suppressed burn-induced ROS production and normalized mitochondrial ultrastructure in mouse skeletal muscle [118].

Pyruvate, a major mitochondrial fuel, is an efficient scavenger or ROS with strong anti-inflammatory activity [119]. The stabilized form of pyruvate, ethyl pyruvate, is a promising agent for treatment of multiple inflammatory organ injuries (for review see [120]). It was reported to decrease ROS production in the pancreatic islet cells of severely burned mice [45]. It was shown to increase the viability of the ischemic zone of burn wounds [121] and alleviate burn-induced lung injury [122].

The efficiency of antioxidant agents in burn injury treatment was demonstrated in several other publications. Thus, Wen et al. [24] found that chemical compound Oltipraz, which activates transcription factor Nrf2, the inducer of antioxidant enzymes, ameliorates cardiac dysfunction in severely burned mice. In an early study by Zang et al [123], the antioxidative cocktail of vitamins decreased mitochondrial damage in myocardium and improved cardiac function in severely burned rats. Fenofibrate, a pharmacological compound suppressing mitochondrial damage and ROS generation [124], increased mitochondrial ATP production and maintained normal levels of cytochrome c oxidase and citrate synthase activities in the skeletal muscle of children who were victims of severe burn [125]. The inhibitor of mitochondrial calcium uniporter, ruthenium red, which suppresses ROS production [126], improved the mitochondrial respiratory control rate and production of ATP in rat cardiomyocytes after severe burn [127]. Metformin that was shown to inhibit ROS release from mitochondria [128] prevented the burn-induced browning of mouse white adipose [129].

Kaneki et al. [130] found that burn injury increased protein farnesylation in mouse skeletal muscle. In the following study [23], they demonstrated that an inhibitor of farnesyltransferase suppressed the burn-induced ultrastructural alteration of muscle mitochondria and prevented the impairment of mitochondrial respiratory supercomplex assembly.

Wen and co-authors [35] found that sildenafil, the inhibitor of cGMP-specific diestherase type 5 (PDE5), applied to severely burned rats, restores mitochondrial function and biogenesis in myocardium. The increase in the cGMP level by PDE5 results in activation of cGMP-dependent protein kinase PKG, which leads to a decrease in intracellular calcium. One can suggest that by inhibiting PDE5 sildenafil prevents the burn-induced release of calcium from ER and thus protects mitochondria. Sildenafil was shown to attenuate burn-induced fibrogenesis and inflammation in rat heart and preserve cardiac function [131]. It improves burn wound healing [132,133,134] and decreases burn-induced skeletal muscle loss [135], liver and kidney injury [136] and acute lung injury [137].

Thus, many compounds normalizing mitochondrial function and suppressing ROS production show potential to be applied for burn treatment. To this list should be added the antifibrinolytic agent tranexamic acid (TXA). This inhibitor of plasmin maturation, widely used to decrease blood loss after hemorrhagic trauma and surgical operations, also decreases edema and SIRS (for review see [138]). Our group found that in vitro TXA strongly enhances mitochondrial respiration and ATP production [139]. When applied to severely burned mice, TXA suppressed the burn-induced increase in the mtDNA level in the bloodstream and invasion of macrophages to the lungs [140]. In the following experiments with severely burned rats, we found that TXA suppresses burn-induced SIRS and lung endothelium leakage and improves the healing of burn wounds [141]. Interestingly, we found that the anti-inflammatory effects of TXA could be independent of its effect on plasmin production. Indeed, TXA suppresses the lipopolysaccharide-induced increase in TNFα and IL1α expression both in wildtype and plasminogen knockout mice [142]. The molecular mechanisms underlying the effects of TXA on mitochondria and alleviation of burn effects are currently under study by our group.

In recent years, numerous articles reported successful use of healthy mitochondria transplantation to treat some diseases and traumas (for review see [143,144]). Many of these studies use mitochondria derived from stem cells. Although exciting and novel, mitochondrial transplantation requires a cautious approach considering the potent proinflammatory effects of mitochondria-derived DAMPs. In addition, it remains to be determined how to most efficiently direct mitochondrial transplantation to treat the generalized organism dysfunction caused by severe burn.

In summary, a large number of mitochondria-targeted molecules have shown their efficiency to treat burn-induced organism dysfunctions (Table 2).

## 8. Conclusions

Burn injury results in massive mitochondrial dysfunction in many organs, including those distant from the burn site. Apparently, the initial factors inducing mitochondrial damage are DAMPs released from burn-injured tissue. A significant role of mitochondrial damage is also played by the catecholamines released to the bloodstream after burn. Over the course of the time, mitochondrial dysfunction is further exacerbated by the massive production of proinflammatory cytokines. The loss of respiratory coupling in mitochondria and massive ROS production play critical roles in the development of burn-related edema, SIRS, muscle wasting, hypermetabolism and adipose browning. Thus, mitochondria are a promising target to develop novel approaches to burn injury treatment.

## Figures and Tables

**Table 1:** Effects of severe burn injury on the mitochondria of different organs.

Organ/Tissue	Species	Dysfunction	References
muscle	human	Reduced oxidation of palmitate and pyruvate	[26]
muscle	mouse	Reduced mitochondrial respiration and increased uncoupling of respiration, and ATP production	[28]
muscle	human	Enhancement of mt-UPR pathway activity	[29]
heart	rat, mouse	Decreased production of ATP in mitochondria	[22,35]
heart	rat	Decreased activity of mitochondrial electron transfer complexes	[35]
heart	porcine	Decreased activity of mitochondrial electron transfer complexes and increased uncoupling	[36]
heart	mouse	Decrease in mtDNA integrity	[37]
lung	mouse	Decrease in mtDNA integrity	[37]
liver	mouse	Decreased mitochondrial respiration and increased uncoupling	[38]
liver	rat	Suppression of cytochrome c and b activities	[39]
liver	rat	Increased ROS production in mitochondria	[40]
liver	mouse	Decrease in mitochondrial respiration and ATP production	[37]
liver	mouse	Decreased fatty acid β-oxidation	[41]
intestine	rat	Increased mitochondrial uncoupling	[43]
pancreas	mouse	Increased ROS production, suppression of electron transfer complex III activity	[45]
adipose	mouse and human	High expression of UCP1, enhancement of uncoupled mitochondrial respiration	[46,47,49]

**Table 2:** Improvement of severe burn effects by mitochondria-targeted compounds.

Agent	Characteristic	Effects in Burned Organism	References
SS-31 peptide	Mitochondria-targeted antioxidant	Decreased ER stress Restoration of mitochondrial enzyme expression Restoration of mitochondrial ATP production and coupling Decrease in mtDNA release and suppression of mtDNA-STING signaling Amelioration of hepatic injury	[109,110,111]
Mito_TEMPO	Mitochondria-targeted antioxidant	Alleviation of cardiac dysfunction Decrease in cardiac inflammation and fibrogenesis Decrease in ROS in myocardium	[112]
ASMq	Natural herb compound	Suppression of burn wound progression Decrease in oxidative stress and mitochondria-related apoptosis	[113]
Taurine	Non-essential amino acid	Protection of mitochondrial enzymes activity Promotion of burn wound healing Anti-inflammatory effect	[115,116,117]
Q10 coenzyme	Mitochondrial ECT cofactor	Decreased ROS production Normalization of mitochondrial ultrastructure	[118]
Pyruvate	Alpha-keto acid	Decreased ROS production Improvement of burn wound ischemic zone Alleviation of lung injury	[45,121,122]
Oltipraz	Inducer of Nrf2 expression	Amelioration of cardiac dysfunction	[24]
Fenofibrate	Antioxidant	Increase in ATP production and mitochondrial enzyme activity	[125].
Ruthenium red	Inhibitor of mitochondrial calcium uniporter	Improvement of mitochondrial respiration and ATP production	[127]
Metformin	Biguanide, anti-hyperglycemic agent	Prevention of burn-induced adipose browning	[129]
FTI	Farnesyltransferase inhibitor	Normalization of mitochondrial ultrastructure and mitochondrial respiratory supercomplex assembly	[23]
Sildenafil	PDE5 inhibitor	Restoration of mitochondrial biogenesis and function Alleviation of cardiomyopathy Improvement of burn wound healing Decrease in skeletal muscle loss Decrease in kidney, liver and lung injury	[35,132,133,134,135,136,137]
Tranexamic acid	Synthetic amino acid, analog of lysine; anti-fibrinolytic agent	Decrease in mtDNA release Decrease in macrophage and neutrophil infiltration in lungs Decrease in vessel leakage in lungs Improvement of burn wound healing	[140,141]

## Data Availability

No new data were created or analyzed in this study. Data sharing is not applicable to this article.
